# Supplementary figures and images for: Prediction of poor outcome after hypoxic-ischemic brain injury by diffusion-weighted imaging: A systematic review and meta-analysis
Source: PLoS One. 2019 Dec 27;14(12):e0226295. doi: 10.1371/journal.pone.0226295 (PMC6934311; doi:10.1371/journal.pone.0226295)

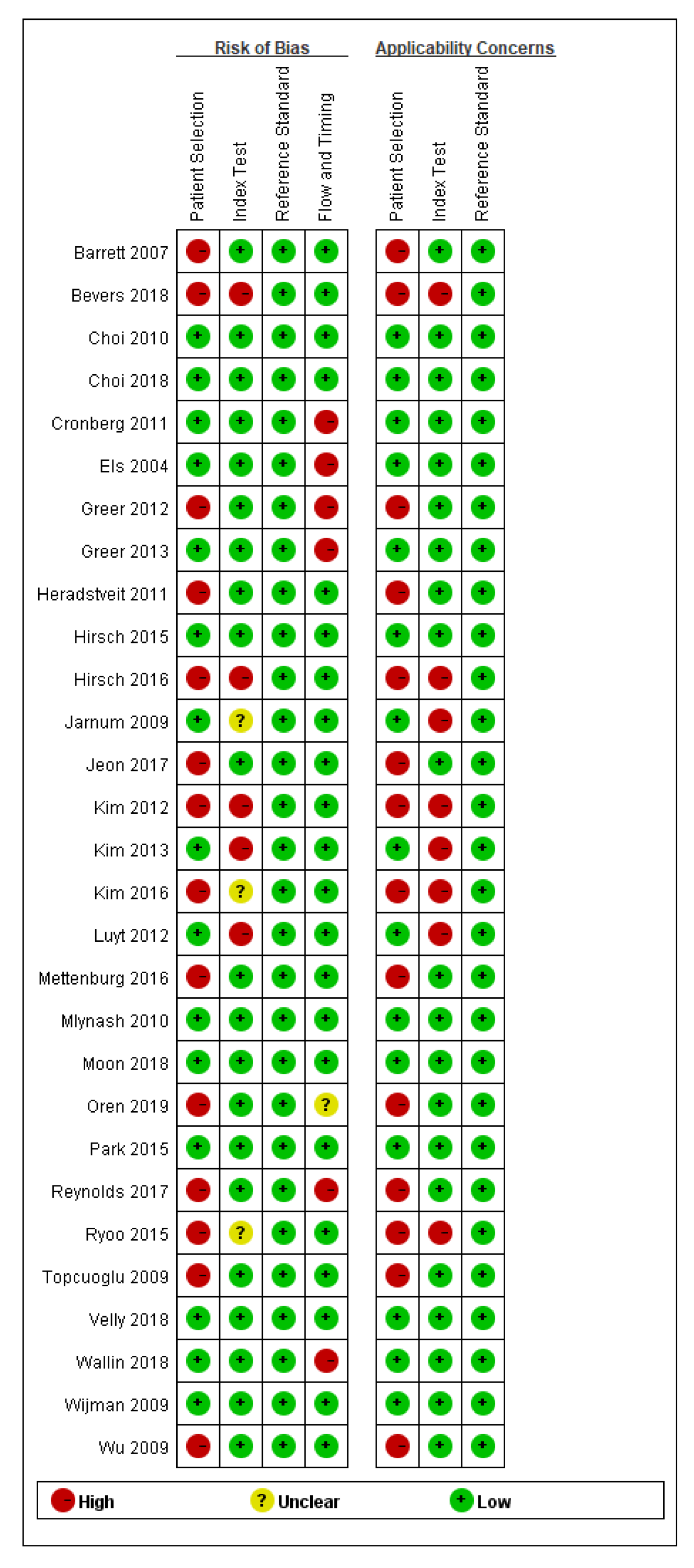

Supplement: S1 Fig — Review authors' judgements about each domain for each included study. (TIF) [file pone.0226295.s002.tif]

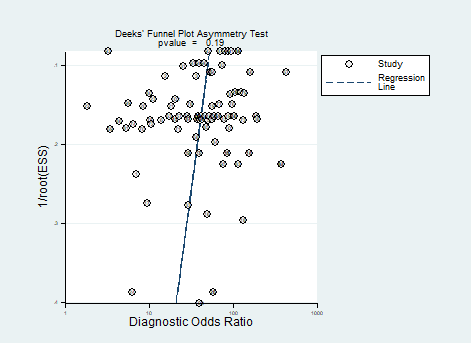

Supplement: S2 Fig — (TIFF) [file pone.0226295.s003.tiff]
