# Supplementary material for: Prediction of poor outcome after hypoxic-ischemic brain injury by diffusion-weighted imaging: A systematic review and meta-analysis
Source: PLoS One. 2019 Dec 27;14(12):e0226295. doi: 10.1371/journal.pone.0226295 (PMC6934311; doi:10.1371/journal.pone.0226295)
Supplement: Attachment — Submitted filename: Response to authors.docx [file pone.0226295.s004.docx]

Review of “*Prediction of neurological outcome after hypoxic-ischemic brain injury by diffusion weighted imaging: A systematic review and meta-analysis*”

The authors gave a review and meta-analysis of different diffusion weighted imaging studies predicting neurological recovery in HIBI patients. They find that DWI has a high diagnostic accuracy, but clinical application is limited due to the high variety in study design of the analyses articles.

Although diagnostic properties for HIBI patients are of great interest to clinicians, the manuscript in its current format has, in my opinion, limited added value for clinical practice. I would therefor advice the authors to revise the manuscript, taking into account the following recommendations:

Major revisions

1. Although the authors have reviewed many studies on DWI in postanoxic coma, a link to pathophysiology and clinic appears to be missing. What kind of processes are visualized with DWI imaging and what underlying pathophysiological mechanisms are visualized using the different DWI techniques? Furthermore in the discussion it is found that different areas of the Brain are more or less sensitive for DWI analysis. Here I am also missing a pathophysiological explanation of these results.
2. From the introduction, the importance of the manuscript does not become really clear. I would recommend the authors to provide more information about the current prognosticators in clinical practice and their prognostic properties. This will indicate why more prognostic parameters are needed and allows the readers to connect the results of this meta-analysis to current clinical practice. The additional value of a meta-analysis could be shown by mentioning earlier studies comparing imaging between patients with good and poor outcome.
3. As the authors state in their discussion, the included studies use highly variable study designs and I wonder if it is fair to pool all these studies. The current analysis compares studies on visual DWI, quantitative ADC maps and even one DTI study. The authors perform a heterogeneity test and conclude that the studies included are highly heterogeneous. I think that was to be expected beforehand and would like to see how heterogeneous the studies are when divided by study design. I would also advice the authors to describe in the manuscript why they chose to pool the different study designs.
4. Some studies appear to be missing to me. For example Velly L, Perlbarg V et al. Lancet neurology 2018 and Sair HI, Hannawi Y, et al. Radiology 2017
5. The definition for poor outcome differs in the various studies, resulting in patients with a CPC score of 3 being in both outcome groups in this meta-analysis. This is also a likely course for heterogeneity between the different studies. The authors state that they have explored this factor of heterogeneity in their results section, but I don’t see the results for this and various other factors explored.
6. The authors use the “ shoulder-arm” shape of the ROC curve to indicate a “threshold” effect. Both term are vague terms that I would like to see explained to the readers to avoid confusion. I checked the provided reference 29 (Kim, Lee et al., Korean J Radiol 2015) for an explanation, but the term “ shoulder-arm effect” is never mentioned here. Please provide the correct reference for explanation.

Minor revisions

- I am confused by some references used throughout the text. The authors should pay more attention to their references
  - P4, last part of introduction: reference 19-23 are given to show that DWI was inconsistent among the different studies. Why not use all studies used in the meta-analysis, since this is actually one of the results?
  - P16, reference 59 is given as a proof that DWI changes are correlated with poor outcome. This has also been shown by the results, why mention this sole reference here?
  - P 17, reference 60 is mentioned as a reference that proofs the reliability of the semi-quantitative scoring technique. However, this is an infant study and should not be used here without clarification.
  - P18, Why is reference 61 is also a study using DWI in postanoxic coma. Why is this study not included in the analysis?
  - P19, reference 18 is mentioned as a “ more recent study” that solves selection bias. However, this seems to be one of the oldest studies included in this manuscript.
- In the introduction, I am missing the EEG as one of the established predictors of post anoxic coma (Hofmeijer et al. Neurology 2015).
- In the discussion, the authors conclude that “ future well designed, large-scale studies should be carried out to confirm the best positive index”. As a clinical researcher, I would like the authors to give more specific recommendations of how these studies should look like
- Heradsveit et al (2011) have shown that ADC values correlate with body temperature. The authors do not find that treatment with hypothermia affect DWI measurements. Since the authors claim already in their introduction that DWI is not affected by hypothermia, I would like to see some discussion on the phenomenon described by Heradstveit et al.
